# Whole Corn Germ as an Energy Source in the Feeding of Feedlot Lambs: Metabolic and Productive Performance

**DOI:** 10.3390/ani12101261

**Published:** 2022-05-14

**Authors:** Camila de O. Nascimento, Douglas dos S. Pina, Stefanie A. Santos, Maria L. G. M. L. de Araújo, Luis G. A. Cirne, Henry D. R. Alba, Thomaz C. G. C. Rodrigues, Willian P. Silva, Carlindo S. Rodrigues, Manuela S. L. Tosto, Gleidson G. P. de Carvalho

**Affiliations:** 1 Department of Animal Science, Universidade Federal da Bahia, Salvador 40170110, Brazil; milaoliver.vet@gmail.com (C.d.O.N.); douglas.pina@ufba.br (D.d.S.P.); stefanie.alvarenga@ufba.br (S.A.S.); mlaraujo@ufba.br (M.L.G.M.L.d.A.); henry.ruiz@ufba.br (H.D.R.A.); thomaz.guimaraes@ufba.br (T.C.G.C.R.); willianzoo@yahoo.com.br (W.P.S.); carlindo.rodrigues@ufba.br (C.S.R.); mtosto@ufba.br (M.S.L.T.); 2 Institute of Biodiversity and Forestry, Universidade Federal do Oeste do Pará, Santarém 68040255, Brazil; luis.cirne@ufopa.edu.br

**Keywords:** by-product, linoleic acid source, ruminant nutrition, sheep

## Abstract

**Simple Summary:**

By-products are the focus of many ruminant nutrition studies in recent decades. These alternative feeds show different concentrations of ideal nutrients for feeding ruminants. These are generally less expensive than common ingredients used in traditional diets (soybean meal and corn). From the corn industry we obtain the by-product whole corn germ (WCG). The highest lipid content of corn is found in WCG (85%), which makes it useful for increasing the energy density of diets. This research aims to evaluate the inclusion in the diet of different levels of WCG, an energy source, on the metabolic and productive behavior of feedlot lambs. Although some changes in nutrient intake and digestibility were observed, the inclusion of WCG in the diet did not promote changes in nitrogen retention, productive performance, and blood parameters. In this way, the use of WCG up to 120 g/kg DM, in the total diet, is recommended as an alternative source of energy for feedlot lambs.

**Abstract:**

The aim of this study was to evaluate the dietary inclusion (0, 30, 60, 90, and 120 g/kg DM) of whole corn germ (WCG), an energy source, on the metabolic and productive performance of feedlot lambs. To this end, two complementary experiments were carried out. In Experiment I, we tested the effects of WCG inclusion levels on the metabolism of 10 uncastrated Santa Inês male lambs, which were distributed into two 5 × 5 Latin squares design. Non-fibrous carbohydrates digestibility decreased (*p* = 0.01), whereas ether extract digestibility increased (*p* < 0.01) with the inclusion of WCG. Retained nitrogen did not change (*p* = 0.99) with the WCG inclusion. In Experiment II, we tested the effect of WCG inclusion levels on the production performance of 40 uncastrated Santa Inês male lambs, in a completely randomized design. There was a reduction in the intake of nutritional components (*p* < 0.05), except EE (*p* < 0.01), which increased with the increasing WCG levels. Production performance and blood parameters did not change. Based on the observed metabolism, performance, and feed efficiency, the use of WCG at up to 120 g/kg DM, in the total diet, is recommended as an alternative energy source for feedlot lambs.

## 1. Introduction

When used properly, the inclusion of high-fat ingredients in ruminant diets can be a strategy to increase their energy density as well as to supply essential fatty acids and fat-soluble vitamins. In addition, this practice can increase the efficiency of use of metabolizable energy for growth by reducing the heat increment [1]. The sheep are animals that adapt quickly and efficiently to dietary challenges. There exists a diversity of plant products and by-products rich in essential fatty acids, such as linoleic acid, a precursor of conjugated linoleic acid (CLA), which is found in large concentrations in corn grains [2].

Corn is an increasingly expensive ingredient due to competition for its use in the human diet, industry, and animal feed. On the other hand, the industrialization of corn generates large and varied quantities of products and by-products, which have nutritional potential for animal feed. A highlighted example of such by-products is whole corn germ (WCG) [3].

Whole corn germ is obtained from the wet degermination of the corn grain [4] and is the result of the grinding of the grain, germ, tegument, and starch particles [5]. Due to its composition, WCG appears to be a by-product with the potential to replace other ingredients in ruminant diets. The chemical composition of WCG includes a crude protein content of 10–15% [6,7], ether extract levels of 15–44% [6,8], and a linoleic acid content between 44 and 56% [9,10], which characterize it as an important source of energy.

Therefore, the use of this by-product in the diets of feedlot lambs may constitute a new alternative energy source to replace traditional high-cost ingredients such as corn. Thus, we have hypothesized that, in view of its nutritional characteristics, WCG can be used partially in lamb diets as an energy source and contribute to the supply of essential fatty acids, such as linoleic acid, without decreasing the animals’ feed efficiency or production performance.

Two simultaneous experiments were therefore developed to evaluate the effects of including WCG on the intake, digestibility, nitrogen balance, blood parameters, feeding behavior and production performance of feedlot lambs.

## 2. Materials and Methods

### 2.1. Location and Ethical Considerations

The experiments were approved by the Ethics Committee on Animal Use at the School of Veterinary Medicine and Animal Science, Federal University of Bahia (approval no. 70/2018), and followed the guidelines established by the National Council for the Control of Animal Experimentation (CONCEA).

Both experiments were carried out on the Experimental Farm of São Gonçalo dos Campos, which belongs to the Federal University of Bahia, in the municipality of São Gonçalo dos Campos—BA, Brazil.

All animals were housed in individual covered stalls with suspended, slatted wooden floors, measuring 1.2 m^2^ (1.2 × 1.0 m). The stalls were equipped with drinkers and feeders that provided ad libitum access to water and the experimental diets. The animals were identified, treated against endo- and ectoparasites and vaccinated against clostridial infections and rabies at the start of the experiment.

In both experiments, the lambs were fed sorghum silage (*Sorghum bicolor* (L.) *Moench*) as roughage and a concentrate composed of ground corn, soybean meal, urea, a specific mineral mixture for sheep and WCG, at a roughage:concentrate ratio of 50:50 (DM basis) as described in a previously published manuscript [9]. The diets were formulated so as to meet the requirements of lambs with an estimated average daily gain of 200 g, in accordance with the recommendations of the National Research Council [11]. The diets were fed twice daily (09.00 and 16.00 h), so as to allow 10% refusals.

### 2.2. Experiment 1

#### 2.2.1. Animals, Experimental Design and Diets

Ten non-castrated Santa Inês male lambs, at approximately four months of age, with an average body weight of 20.4 ± 3.6 kg (mean ± standard derivation), were distributed into two 5 × 5 Latin squares represented by five animals and five experimental diets (WCG levels of 0, 30, 60, 90, or 120 g/kg DM). The experiment was divided into five sub-periods (15 days of adaptation to the diets and five days of data and sample collection).

#### 2.2.2. Nutrient Intake

To evaluate the intake of nutritional components, the amounts of feed supplied and refusals were recorded daily. Samples of the diets and refusals were collected weekly and stored in plastic containers and kept in a freezer at −20 °C. Subsequently, the samples were pre-dried in a forced-air oven at 55 °C for 72 h and ground in a Wiley knife mill with 1-mm and 2-mm sieves, packed and stored until laboratory analysis. The intake of each nutritional component (g) was calculated as the difference between the amounts of a given nutrient in the feed supplied and in refusals.

#### 2.2.3. Fecal Collection and Nutrient Digestibility Trial

The digestibility trial was conducted between the 14th and 20th days of each experimental period. The first two days were used for the animals to adapt to the collection bags, followed by five subsequent days of total fecal collection. Feces were collected directly into collection bags, twice daily (11.00 and 16.00 h). Then, the total fecal production of each animal was recorded and aliquots of approximately 10% of the total collected were removed, stored in individual plastic bags, labeled, and frozen at −20 °C. During the digestibility trial, samples of feed and refusals were collected daily.

The digestibility coefficient (DC) of the nutritional fractions was calculated by the following formula:

DC = [(Nutrient intake(g) − Nutrient in feces(g))/Nutrient intake(g)] × 100,

where Nutrient intake(g) = Nutrient supplied(g) − Nutrient in refusals(g).

#### 2.2.4. Urine Collection and Nitrogen Balance Trial

On the 16th and 17th days of each experimental period, urine collection funnels were attached to the animals for their adaptation. Between the 18th and 20th days, total urine collection was performed using hoses attached to the funnels, which conducted the urine to a plastic container with 100 mL of 20% H_2_SO_4_ (*v*/*v*) [12]. At the end of 24 h, the urine pool was weighed, homogenized, and filtered through two layers of gauze tissue and an aliquot of 10% of the total collected was removed and stored at −20 °C until analysis.

The amount of nitrogen in the samples of feed, refusals, feces, and urine was determined following the methods proposed by the Association of Official Analytical Chemists (Method 968.06) [13].

The apparent nitrogen balance (NB) was calculated using the following formulas:

NB or Nretained = Nintake − (Nfeces + Nurine);

Nabsorbed = Nintake − Nfeces; and

Nintake = Nsupplied − Nrefusals.

### 2.3. Experiment II

#### 2.3.1. Animals, Experimental Design and Diets

Forty uncastrated Santa Inês male lambs at approximately four months of age, with an average body weight of 22.1 ± 4.0 kg (mean ± standard derivation), were allocated to five experimental treatments (diets with WCG levels of 0, 30, 60, 90, or 120 g/kg DM), using eight replicates (animals), in a completely randomized experimental design. The total duration of the experiment was 82 days, which consisted of 15 days of adaptation to the environment, management, and diets plus the actual experimental period of 67 days.

#### 2.3.2. Nutrient Intake

The collection and processing of samples and data to evaluate the intake of nutritional components in Experiment II were the same as described for Experiment I. The nutritional components in the actually consumed diet were calculated by dividing the intake of each nutrient by the DM intake (DMI) and multiplying the result by 100.

#### 2.3.3. Feeding Behavior

On the 25th and 40th days of the experimental period, all animals were observed visually for the assessment of feeding behavior. Observations were performed at 5-min intervals over 24 h by nine trained observers, to calculate the time expended on the feeding, rumination, and idling activities [14]. During the night, the area was kept under artificial light two days before data collection. The observers did not know the distribution of the treatments and were positioned to disturb the lamb behavior as little as possible. Using a digital stopwatch, on the same day, the number of rumination chews and the time expended to ruminate each cud were measured. For this evaluation, three cuds were observed in three different periods of the day (10.00–12.00, 14.00–16.00, and 19.00–21.00 h) to determine the average number of rumination chews and the time expended per cud. During the night, the environment was kept under artificial lighting.

Feeding efficiency (FE), rumination efficiency (RE), the number of cuds ruminated per day (CRD), and the number of rumination chews per day (RChD) were calculated as described by Polli et al. [15] and Bürger et al. [16], as follows:

FE (g DM/h) = DMI (g DM/day)/Feeding time (h/day);

RE (g DM/h) = DMI (g DM/day)/Rumination time (RT, h/day);

CRD (n/day) = RT (s/day)/Chewing time per ruminated cud (s/cud);

RChD (n/day) = CRD × Number of rumination chews per cud (n/cud)

#### 2.3.4. Blood Metabolites

On the 60th day of the experimental period, a 10-mL blood sample was collected in Vacutainer tubes containing anticoagulant (EDTA) four hours after the first feeding. The samples were immediately centrifuged at 3500 rpm for 15 min to obtain the plasma. Subsequently, the obtained plasma was transferred to the labeled Eppendorf tubes and stored at −20 °C for further analysis.

Plasma cholesterol and triglyceride concentrations were determined by the liquid enzymatic assay technique. The total plasma proteins and albumin were measured by colorimetry, and the globulin content was calculated as the difference between the total protein and albumin contents, with results expressed in g/dL.

The plasma concentrations of the enzymes for the evaluation of liver metabolism, namely, aspartate-aminotransferase (AST), alanine-aminotransferase (ALT), and gamma-glutamyl transferase (GGT), were measured by the kinetic system.

Plasma metabolite analyses were carried out using commercial kits (Doles^®^), with readings taken by a semi-automatic spectrophotometer (SBA 200^®^, CELM, São Caetano do Sul, Brazil) at the respective wavelengths at the Animal Nutrition Laboratory (LANA), Federal University of Bahia (UFBA).

#### 2.3.5. Performance

For the assessment of performance, the lambs were weighed on the first and last experimental days, after a 16-h fast of solid feed. Total weight gain (TWG), average daily gain (ADG), and feed efficiency (FE) were calculated.

#### 2.3.6. Laboratory Analysis

In Experiments I and II, the dry matter (DM; method 934.01), ash (method 942.05), crude protein (CP; method 968.06) and ether extract (EE; method 920.39) contents were analyzed as recommended by the Association of Analytical Chemists [13]. The neutral (aNDF—NDF assayed with a heat stable amylase and expressed inclusive of residual ash) and acid (ADF) detergent fiber contents were determined according to Mertens [17] and Van Soest et al. [18], respectively. Lignin was determined using method 973.18 of AOAC [19], by treating the ADF residue with 72% sulfuric acid. The neutral (NDIP) and acid (ADIP) detergent insoluble protein contents were determined according to Licitra et al. [20].

The non-fibrous carbohydrate (NFC) content of the diets was calculated using the equation proposed by Hall [21]: NFC = 100 − ((%CPdiet − %CPurea + % Ureadiet) + %EE + aNDF + %Ash).

The total digestible nutrients (TDN) of the diets were estimated using the specific equations for small ruminants proposed by da Cruz et al. [22]. The metabolizable energy was estimated as proposed by Weiss and Tebbe [23].

#### 2.3.7. Fatty Acid Profile

The lipids composition was determined by converting the lipid extracts to fatty acid methyl esters (FAME). Immediately, the FAME sample was processed following the methodology of O’Fallon et al. [24].

The procedures performed to synthesize FAME and identify the fatty acid composition of diets are described in Nascimento et al. [9], following the methods of Kramer et al. [25] and Bravo-Lamas et al. [26].

Fatty acid methyl esters were quantified following the equation proposed by Sukhija and Palmquist [27]: [(total area of the peaks—area of the internal standard)/area of the internal standard) × (concentration of the internal standard/weight of the lyophilized sample)]. Finally, the fatty acid profile was expressed in mg of fatty acids per kg of diet (mg/kg).

### 2.4. Statistical Analysis

To determine the sample size of the present study, the proc GLMPOWER was used and to randomize the animals within the treatments, the PROC PLAN; both procedures of SAS 9.4 software. Results were subjected to statistical analysis in a double Latin square design (5 × 5) (Experiment I) and in a completely randomized design (Experiment II) using the PROC MIXED procedure of SAS 9.4, respectively. The following models were applied:

Yijkl = µ + SLi + A(LSi)j + Pk + GIl + εijklm,

where Yijkl = dependent variable; µ = overall mean; LSi = random effect of Latin square (i = 1 to 2); A(LSi)j = random effect of animal within Latin square (j = 1 to 5); Pk = random effect of period (k = 1 to 5); GIl = fixed effect of WCG level (l = 0, 30, 60, 90 or 120 g/kg DM), εijklm = random error assumed NID~(0, σ2).

Yij = µ + GIi + εij,

where Yij = dependent variable; µ = overall mean; GIi = fixed effect of WCG level (I = 0, 30, 60, 90 or 120 g/kg DM); and εij = random error assumed NID~(0, σ2).

In both models, the effect of WCG inclusion level was evaluated by fitting orthogonal polynomial contrasts to evaluate the linear (−2 −1 0 +1 +2) and quadratic (+2 −1 −2 −1 +2) effects. For all evaluations, a ≤5% probability level for type-I error was considered significant and ≤10% as a trend.

## 3. Results

### 3.1. Experiment I

#### 3.1.1. Nutrient Intake and Digestibility

Dietary inclusion of WCG induced a linear decrease in the intake of DM (*p* < 0.01) and OM (*p* < 0.01); as well as a linear decrease trend in the aNDF intake (*p* = 0.09). On the other hand, the CP (*p* = 0.04) and NFC (*p* < 0.01) intakes responded quadratically, with a minimum CP intake of 188.9 g/day occurring at the WCG level of 82 g/kg DM and a minimum NFC intake of 332.1 g/day seen at the WCG level of 104 g/kg DM. Ether extract intake, in turn, increased (*p* < 0.01) with the WCG levels (Table 1).

The increasing levels of WCG in the diet did not compromise (*p* > 0.05) the digestibility of nutritional components, except EE, whose digestibility increased (*p* < 0.01); NFC, whose digestibility decreased linearly (*p* < 0.01); and TDN, whose digestibility increased linearly (*p* = 0.05) (Table 1).

#### 3.1.2. Nitrogen Balance

The increasing WCG levels in the lambs’ diet induced a quadratic response (*p* = 0.03) from N intake and N excretion in the feces (*p* = 0.02). The minimum N intake was 30.3 g/day, which occurred at the WCG level of 87 g/kg DM level, whereas the minimum fecal N content was 9.9 g/day at 88 g WCG/kg DM. Nonetheless, the dietary inclusion of WCG did not affect urinary N, retained N, or absorbed N (*p* > 0.05) (Table 2).

### 3.2. Experiment II

#### 3.2.1. Intake of Nutritional Components

There was a reduction (*p* < 0.01) in the intakes of all nutritional components, except EE, which increased with the dietary WCG levels (*p* < 0.01). However, the actually consumed amounts of EE (*p* < 0.01), aNDF (*p* < 0.01) and NFC (*p* < 0.01) increased (Table 3).

#### 3.2.2. Blood Metabolites

Total plasma proteins increased (*p* = 0.03) with the increasing levels of WCG in the diet. However, the albumin (*p* = 0.01) and globulin (*p* = 0.01) contents and consequently, the albumin:globulin ratio (*p* < 0.01), showed a quadratic response, with the maximum albumin content (2.5 mg/dL) occurring at the WCG level of 29 g/kg DM. In contrast, the minimum globulin content (3.5 mg/dL) occurred at the WCG level of 30 g/kg DM. The highest albumin:globulin ratio observed was 0.72 mg/dL, at the WCG level of 28 g/kg DM. The enzyme AST increased with the amount of WCG added to the diet (*p* < 0.01) (Table 4). Furthermore, a quadratic trend (*p* = 0.05) was observed in the concentration of cholesterol in blood, decreasing after the inclusion of WCG of 56 g/kg DM.

#### 3.2.3. Feeding Behavior and Performance

There was an increase (*p* < 0.01) in rumination time and a reduction (*p* < 0.01) in idle time (Table 5) as the WCG levels in the diet were increased. In the evaluation of rumination chews, the number of chews per day increased (*p* < 0.01), whereas the amount of DM (g) per cud decreased (*p* < 0.01) in the lambs that received higher levels of WCG. Feeding and rumination efficiencies in g DM/h decreased (*p* < 0.01), whereas feeding efficiency in g NDF/h increased (*p* = 0.03) with the dietary inclusion of WCG. The time expended per rumination periods was longer (*p* = 0.01) in animals fed the diets with higher levels of WCG (Table 5).

The inclusion of WCG in the feedlot lambs’ diet did not influence (*p* > 0.05) the production parameters that defined animal performance (Table 6). However, a trend (*p* < 0.10) of decreasing performance (TWG, ADG) was observed in lambs fed increasing levels of whole corn germ.

## 4. Discussion

### 4.1. Experiment I

The decreasing DM intake in response to the increasing WCG levels in the diet can be explained by the EE and polyunsaturated fatty acid contents of this by-product. Polyunsaturated fatty acids that reach the intestine are rapidly absorbed and oxidized in the liver, transmitting inhibitory signals to the hypothalamus and thus triggering satiety [28].

High levels of EE can alter rumen metabolism and hinder the degradation of other nutritional components [29], which is mainly due to the toxicity caused by unsaturated fatty acids to rumen bacteria. However, the EE provided by WCG did not affect the digestibility of nutritional components in the present study. This fact suggests that the polyunsaturated fatty acids present in WCG [9] can probably be protected from the ruminal biohydrogenation process [30] and thus be rapidly absorbed by the intestine without interfering with the animals’ ruminal metabolism. Furthermore, the increased EE content of the diets provided by the inclusion of WCG resulted in a greater intake of this nutrient, in addition to greater digestibility.

As expected, N intake and fecal excretion followed the same quadratic behavior shown by CP intake. However, the inclusion of WCG in the diet did not influence N recycling, and retained N remained similar between the treatment groups. The observed values are in agreement with those described in other studies that tested diets with similar nutritional profiles [31,32,33].

### 4.2. Experiment II

Studies using WCG to replace corn meal in sheep diets have shown a reduction in DM intake at higher WCG levels [9,34,35]. As discussed earlier, when oxidized in the liver, the high EE content and the polyunsaturated fatty acid content of WCG [9] activate satiety, reducing DM intake. However, EE intake was higher in the groups fed the diets with higher levels of this ingredient. Nonetheless, the higher values for actually consumed fractions of EE, aNDF, and NFC can be explained by the levels of these nutritional components in WCG [9], which shows that the highest proportions of total DM intake consisted of these components.

The diets formulated with higher levels of WCG had higher aNDF content [9]. Coupled with the decreasing DM intake, this fact explains the increase in rumination time, which also resulted in a higher number of chews. Oliveira et al. [36] observed a similar situation.

As a compensatory effect to the possible effect of EE on the digestion of fiber, the lambs fed diets with higher levels of WCG showed greater feeding and rumination efficiencies in NDF, as also demonstrated in the study led by Miotto et al. [37]. According to Mertens [38], factors related to the NDF present in the diet, such as particle size, in addition to its digestible and indigestible fractions, can influence feeding behavior, culminating in changes in the feeding, rumination, and idle times. On the other hand, with the reduction in DM intake, the lambs’ feeding and rumination efficiencies in DM also declined.

Despite the reduction in the intake of some nutritional components, the dietary inclusion of WCG did not influence the weight gain or feed efficiency of the animals, whose respective results were 232 g/day and 188 g of gain/kg of DM ingested. Therefore, weight gain was adequate and consistent with a balanced diet. The long-chain fatty acid profile of WCG [9] may have contributed to the higher energy density of the diet. In addition, it may also have conditioned the lambs to make greater use of dietary protein for muscle growth and of dietary energy for physiological maintenance [9,39].

The feed efficiency values found in this study are in line with those considered normal (approximately 200 g of gain/kg of DM ingested) for Santa Inês crossbred lambs [40]. The similar results for feed efficiency between the treatment groups confirm that despite the metabolic changes that affected intake, the animals were efficient in using energy from the actually consumed diets.

The evaluation of blood metabolites is important to check the nutritional and metabolic status of animals. In this respect, we detected an increase in the concentrations of total plasma proteins, which shows that WCG inclusion did not negatively affect protein metabolism. The slight decrease in albumin levels consequently led to a reduction in albumin:globulin ratio. Additionally, the increased levels of AST, an enzyme that indicates the liver profile, suggest mild liver injury, likely due to the high EE content of the diet, since lipid is metabolized in the liver. Due to the fact that albumin is metabolized in this organ, a lesion, even if mild, can compromise the production of this protein [41]. However, the blood metabolite levels found in the current experiment are within the recommended reference values for healthy adult sheep [42,43].

The higher content of EE in the diet limited the DMI of the feedlot lambs in the current study. DMI is one of the main factors affecting the performance of animals [28]. Probably these factors promoted the tendency to a lower performance (FW, ADG, and ADG) of the animals. This theory can be corroborated by the trend observed in the feed efficiency, which indicates that with increasing levels of WCG, more grams of feed are needed to gain 1 kg of weight.

## 5. Conclusions

Based on the metabolic profile, feed efficiency and production performance shown by the animals, the use of whole corn germ at up to 120 g/kg DM is recommended, as an alternative energy source, in the diet of feedlot lambs.

## Figures and Tables

**Table 1:** Intake and digestibility coefficients of nutritional components in lambs fed diets containing whole corn germ.

Item	Inclusion Level of WCG (g/kg DM)					SEM	*p*-Value ^1^	
	0	30	60	90	120		L	Q
Intake (g/day)								
Dry matter	1232.1	1140.0	1003.1	1032.3	1051.3	52.02	<0.01	0.06
Organic matter	1185.1	1096.2	965.0	992.7	1010.1	50.36	<0.01	0.06
Crude protein	227.2	209.5	186.9	189.2	195.5	8.85	<0.01	0.04
Ether extract	40.8	52.7	62.0	76.8	94.4	2.81	<0.01	0.12
Neutral detergent fiber	441.7	419.3	473.3	394.5	394.9	21.15	0.09	0.17
Non-fibrous carbohydrates	478.3	418.4	347.7	336.7	331.3	17.90	<0.01	0.02
Total digestible nutrients	810.5	798.5	692.2	712.0	755.6	45.81	0.19	0.19
Digestibility coefficient (%)								
Dry matter	61.6	65.2	60.6	60.2	61.0	2.07	0.37	0.86
Organic matter	64.1	67.1	62.7	62.5	63.2	1.97	0.32	0.96
Crude protein	63.1	66.4	65.6	65.0	68.1	1.87	0.16	0.99
Ether extract	80.0	85.2	86.8	87.4	89.5	0.77	<0.01	0.02
Neutral detergent fiber	45.9	52.6	48.6	47.5	46.5	3.18	0.70	0.31
Non-fibrous carbohydrates	79.7	79.9	72.8	73.3	73.0	1.77	<0.01	0.34
Total digestible nutrients	65.2	69.8	67.8	68.9	71.4	1.75	0.05	0.88

^1^ L: Linear; Q: Quadratic. Effect significant at *p* ≤ 0.05.

**Table 2:** Intake of nutritional components and fractions actually consumed by lambs fed diets containing whole corn germ.

Nitrogen (g/day)	Inclusion Level of WCG (g/kg DM)					SEM	*p*-Value ^1^	
	0	30	60	90	120		L	Q
Intake	36.4	33.5	29.6	30.5	31.2	1.41	<0.01	0.03
Fecal	13.2	11.1	9.8	10.4	10.1	0.60	<0.01	0.02
Urinary	7.5	5.9	4.5	6.5	6.4	1.12	0.66	0.15
Absorbed	23.1	22.3	19.7	20.3	21.1	1.31	0.15	0.21
Retained	15.6	16.4	15.2	13.8	14.7	1.69	0.41	0.99

^1^ L: Linear; Q: Quadratic. Effect significant at *p* ≤ 0.05.

**Table 3:** Apparent nitrogen balance in lambs fed diets containing whole corn germ.

Item	Inclusion Level of WCG (g/kg DM)					SEM	*p*-Value ^1^	
	0	30	60	90	120		L	Q
Nutrient intake (g/day)								
Dry matter	1232.1	1140.0	1003.1	1032.3	1051.3	52.02	<0.01	0.06
Organic matter	1185.1	1096.2	965.0	992.7	1010.1	50.36	<0.01	0.06
Crude protein	227.2	209.5	186.9	189.2	195.5	8.85	<0.01	0.04
Ether extract	40.8	52.7	62.0	76.8	94.4	2.81	<0.01	0.12
Neutral detergent fiber	441.7	419.3	473.3	394.5	394.9	21.15	0.09	0.17
Non-fibrous carbohydrates	478.3	418.4	347.7	336.7	331.3	17.90	<0.01	0.02
Total digestible nutrients	810.5	798.5	692.2	712.0	755.6	45.81	0.19	0.19
Actually consumed fraction of the diet (%)								
Dry matter	36.4	33.5	29.6	30.5	31.2	1.41	<0.01	0.03
Organic matter	13.2	11.1	9.8	10.4	10.1	0.60	<0.01	0.02
Crude protein	7.5	5.9	4.5	6.5	6.4	1.12	0.66	0.15
Ether extract	23.1	22.3	19.7	20.3	21.1	1.31	0.15	0.21
Neutral detergent fiber	15.6	16.4	15.2	13.8	14.7	1.69	0.41	0.99
Non-fibrous carbohydrates	36.4	33.5	29.6	30.5	31.2	1.41	<0.01	0.03
Total digestible nutrients	13.2	11.1	9.8	10.4	10.1	0.60	<0.01	0.02

^1^ L: Linear; Q: Quadratic. Effect significant at *p* ≤ 0.05.

**Table 4:** Concentration of metabolites and liver enzymes in the blood of lambs fed diets containing whole corn germ.

Item	Inclusion Level of WCG (g/kg DM)					SEM	*p*-Value ^1^	
	0	30	60	90	120		L	Q
Blood metabolites (mg/dL)								
Total proteins	6.10	6.03	5.94	6.31	6.55	0.17	0.03	0.10
Albumin (A)	2.51	2.51	2.47	2.40	2.14	0.05	<0.01	0.01
Globulin (G)	3.59	3.51	3.56	3.90	4.40	0.14	<0.01	0.01
A:G	0.70	0.72	0.69	0.63	0.49	0.02	<0.01	<0.01
Triglycerides	35.0	33.9	34.8	31.7	29.8	3.33	0.27	0.67
Cholesterol	41.6	53.0	49.4	46.9	43.8	3.75	0.88	0.05
Liver enzymes (IU/L)								
Alanine aminotransferase	10.0	9.6	9.6	11.1	9.75	1.48	0.83	0.92
Aspartate aminotransferase	76.4	79.6	87.0	91.8	90.4	4.08	<0.01	0.47
Gamma-glutamyl transferase	54.0	55.8	54.8	46.8	52.9	2.85	0.22	0.88

^1^ L: Linear; Q: Quadratic. Effect significant at *p* ≤ 0.05.

**Table 5:** Feeding behavior of lambs fed diets containing whole corn germ.

Item ^1^	Inclusion Level of WCG (g/kg DM)					SEM	*p*-Value ^2^	
	0	30	60	90	120		L	Q
Feeding time (min/day)	193.8	204.6	200.5	197.6	195.2	9.52	0.89	0.48
Rumination time (min/day)	489.8	510.0	517.4	545.8	551.0	17.40	<0.01	0.89
Idling (min/day)	756.4	725.4	722.1	696.6	693.8	16.55	<0.01	0.58
Chewing (n/cud/day)	703	684	736	712	729	31.30	0.42	0.97
Chewing (n/cud/h)	87.3	80.8	86.0	78.5	80.0	3.60	0.15	0.82
Chewing (s/cud)	41.8	45.4	43.1	46.1	44.4	1.97	0.34	0.46
Chewing (n/day)	29,392	30,594	31,036	32,751	33,054	1044.5	<0.01	0.89
Chewing (g DM/cud)	2.0	1.9	1.7	1.6	1.60	0.09	<0.01	0.48
Feeding efficiency								
g DM/h	440.3	409.2	378.1	327.7	374.4	21.13	<0.01	0.09
g NDF/h	126.6	124.8	133.8	134.2	143.6	6.17	0.03	0.56
Rumination efficiency								
g DM/h	183.8	162.7	145.3	125.0	132.1	6.12	<0.01	0.02
g NDF/h	52.2	49.2	50.5	48.1	50.0	1.67	0.33	0.35
Number of periods (n/day)								
Feeding	14	15	15	14	13	1.03	0.34	0.26
Rumination	29	29	27	30	29	0.99	0.60	0.57
Idling	40	40	38	40	39	1.40	0.64	0.83
Time expended per period (min)								
Feeding	14.0	13.6	13.4	14.2	15.2	0.61	0.13	0.10
Rumination	16.3	18.2	19.1	18.2	19.6	0.77	0.01	0.35
Idling	19.1	18.6	19.0	17.5	18.4	0.87	0.35	0.80

^1^ DM: dry matter; NDF: neutral detergent fiber; ^2^ L: Linear; Q: Quadratic. Effect significant at *p* ≤ 0.05.

**Table 6:** Performance of lambs fed diets containing whole corn germ.

Item	Inclusion Level of WCG (g/kg DM)					SEM	*p*-Value ^1^	
	0	30	60	90	120		L	Q
Initial weight (kg)	26.9	28.0	26.5	25.8	25.8	-	-	-
Total weight gain (kg)	16.3	16.6	15.2	14.3	15.2	0.82	0.08	0.61
Average daily gain (g)	244	248	228	214	226	12.27	0.08	0.61
Feed efficiency (g/kg)	181	180	182	202	196	0.01	0.07	0.82

^1^ L: Linear; Q: Quadratic. Effect significant at *p* ≤ 0.05.
